# Westminster Ophthalmic Hospital

**Published:** 1832-07-01

**Authors:** 


### LXXXV.

### Westminster Ophthalmic Hospital.

A quarterly return of the patients treated in and at this hospital has lately been made to its governors and friends. It shews the number of applicants on the increase, and the only source of regret that it presents, is the circumstance of the inadequacy of the funds to confer all the benefit that could be wished and is solicited. Those funds, however, are now on the increase, and we trust will yet be greatly and sufficiently augmented.

The results of the treatment of the patients that have attended during the period included in the return, have been highly satisfactory; and, by a reference to another part of this report, it will be seen that the treatment adopted in the severest form of purulent inflammation of the conjunctiva, gonorrhœal ophthalmia, has proved as successful as heretofore. In none of those instances where infants have been brought labouring under purulent ophthalmia, prior to disorganization of one or more of the textures of the eye, has there been an unfavourable result. But parents not unfrequently apply with children, who, from neglect, have lost one or both their eyes by this complaint. Since January, children have been brought to the hospital under these unfortunate circumstances.

The cases of catarrhal inflammation have been successfully treated by the early or immediate use of stimulating applications, conjoined, in those instances which seemed to require them, with the application of leeches, or the abstraction of blood by cupping; but the cases requiring such local depletion were not numerous.

In the treatment of inflammations of the cornea, terminating in, or rather causing, interstitial abscess, the exhibition of mercury has been chiefly relied upon. In some cases, where constitutional symptoms demanded it, quinine has been advantageously combined with it. Matter effused into the anterior chamber, and constituting "hypopium," has, in all the cases, been absorbed under the use of the mercury; and local stimulants were resorted to for the treatment of the superficial ulceration of the cornea. All these cases but one ended favourably. In that one instance, ulceration penetrated the cornea, the iris protruded through the ulcerated aperture, and loss of vision is the consequence, the eye being staphylomatous. This is mainly to be attributed to the careless conduct of the parents of the child, in not bringing her to the hospital, so that the proper plan of treatment could not be pursued.

Ulcers of the cornea have been treated by the use of solutions of the nitrate of silver, the ointment of the same, and, in some instances, the application of that caustic in substance. The granulated state of the lids, the result, most frequently, of erysipelatous inflammation, has been treated by the application of the sulphate of copper in substance, rubbed over the granulated surface. Many of these cases, necessarily tedious from their nature, have, however, given way under this method, and the irritation resulting from the condition of the conjunctiva has thus been removed.

Among the patients presenting themselves with scrofulous ophthalmia, there were many that required long and continued treatment; and, of course, one system would not apply to the variety of cases which occurred. In some, local stimulants and the antiphlogistic plan answered best—in others, tonics were required: several were benefited by the internal use of the liquor potassæ, and three or four almost inveterate cases were cured by the exhibition of the ext. hyosciami, after the foregoing methods had failed of success. Keeping the lower lid depressed by the use of adhesive plaister, in one instance, seems to have been beneficial.

Many of the cases of amaurosis were obviously incurable; but several individuals have completely regained their vision, although they had laboured for some time under this disease; in many of the severer cases, relief has been given to urgent or painful symptoms.

Several patients have applied with disorganization of the eye from inflammation, in some cases the consequence of injuries. Such relief was afforded as their particular states would admit of; but in some instances, the derangement of the structure of the eye was so great, that a cessation of local irritation was all the benefit that could possibly be obtained.

The treatment of iritis, has been that usually adopted at this hospital—antiphlogistic remedies—the use of oil of turpentine in some, and of mercury in other cases. Two of these, as will be seen in a more detailed account, were not so successful as the remainder; but there are some peculiar features in these cases which will account in part for the occurrence. Such has been the treatment of the cases contained in the numerical return annexed to this report. The operations for cataract have, with one exception, a case of extraction, been attended with success; but in that instance so much inflammation followed the operation, that the patient will not recover the use of that eye.

| | |
|---|---|
| Operation of Depression ...... | 1 |
| ———— Breaking up the Cataract ... ............... | 6 |
| ———— Extraction ...... | 3 |
| ———— for a capsular Cataract. | 1 |

To be repeated.

Added to this statement is a more detailed report of such cases as possess particular interest. They are extracted from the books which are kept at the hospital, and have all occurred during the last few months. We shall endeavour to select such cases of each of the more important and frequent diseases of the eye, as will display the effects of different or opposite methods of treatment. It is only by comparisons like these, instituted in a spirit of candour and pursued with care, uncoloured by partiality, and as scrupulously correct in the detail as circumstances will permit, that any solid or permanent improvement can be expected in the treatment of these complaints. With every disposition to adhere to the facts, inaccuracies must necessarily creep in, and all that the reporters can promise, is to use their best endeavours to avoid them.

In the following report we shall pursue what appears to be a natural order, giving first the cases of affections of the superficial tunics, and external parts of the apparatus of vision, and then the deeper-seated diseases. Pursuant to this method we will consider seriatim the affections of the conjunctiva—those of the cornea—of the iris—of the lens—and finally that extensive and formidable class of alterations of the deeper tunics, of which amaurosis is the symbol and the representative.

Before we commence the detail of these cases, we venture to indulge in a few observations on the general arrangements of this Institution, and in doing so we reluctantly refrain from offering Mr. Guthrie what cannot justly be termed a compliment, on the indefatigable zeal with which he has raised it to its present station, the talent with which he presides over the department peculiarly his own, and the anxiety which he displays in affording opportunities of practice and instruction to the gentlemen who are studying ophthalmic surgery within its walls. These gentlemen, owing to the opportunities which they enjoy, must necessarily extend to a very wide circle, the advantages derived from the establishment of an Ophthalmic Hospital at the West end of the metropolis.

To return from this digression. The hospital is calculated to accommodate both in and out patients. Owing to the expenses which have been incurred in the erection of the building, but few in-patients can at present be received, but the number of those who attend as out-patients is very considerable. The latter are seen regularly on the days of Mr. Guthrie's attendance, thrice in the week. From 120 to 140 and upwards are usually present. Patients are admitted on application. After the applicant is seen by Mr. Guthrie, he or she is confided to the care of a pupil, who obtains the management of the case under the superintendence, and, if necessary, direction of Mr. Guthrie. Notes of each case, with the record of the treatment are preserved, and if the details are sufficiently interesting they are transferred to the hospital books which are kept for the purpose. Such is a general outline of the management of the institution. It is supported by subscribers of one or two guineas annually, who become by their subscription Governors. We do not solicit eleemosynary bounty for the hospital, yet we may be permitted to observe that it well deserves the encouragement of those anxious for the diffusion of information and advancement of science in the profession, and of all benevolent and humane individuals out of it. If sufficiently supported, it will be the means of materially diminishing the infirmities and consequently the miseries of the poor in its immediate vicinity, as well as of rendering the young men educated in the West end of the metropolis more qualified to execute their professional duties, with credit to themselves, and advantage to the public.

### Affections of the Conjunctiva.

### 1. Catarrhal Ophthalmia.

This, as our readers are aware, is the most common affection of the eye, at least in persons beyond the age of puberty and adults. The redness is usually inconsiderable, at least in the conjunctiva of the globe—there is much epiphora—and the feeling of sand within the palpebræ is very inconvenient. The usual treatment pursued at this hospital, in recent cases, is to give an emetic or purgative, or an emeto-cathartic, as the mixture of the tartarized antimony and the sulphate of magnesia, and drop into the eye a solution of the nitrate of silver, gr. iv. ad ℥j. or introduce the unguentum argenti nitratis. Leeches, cupping, or blisters are rarely used, but occasionally they are so, when the severity of the pain, or the extreme vascularity, or extreme epiphora, appear to require them. This treatment is very successful, and practitioners unaccustomed to or unacquainted with it, at times evince no little surprise at the benefits derived from it. We shall give a few cases in which different applications were adopted.

Case 1.—*Catarrhal Inflammation of both Eyes—Solut. Arg. Nit. used for one—Ung. Arg. Nit. for the other. Cure.*

Ann Andrews, æt. 39, a waistcoat-maker, stout and apparently healthy, applied at the hospital April 2d, 1832. There was catarrhal ophthalmia of either eye, puffiness of the palpebræ, a sensation of great heat, and much epiphora. The right eye had been affected for five days, and worse than the left; this latter had been affected for three days.

*Unguentum argent. nit. oculo dextro. Lotio argenti nitratis,* gr. iv. *ad* ℥j. *oculo sinistro. Magnes. sulph.* ℥ss. *c. Ant. tart.* gr. ij. *hodie.*

3d. *Repet. ung. et lotio.*

4th. Both eyes greatly improved, left the most so. Says that the ointment produces such pain, which continues during the day, that she will not submit to its future employment. Wishes solution to be used to both eyes.

*Appr. lotio oculo utrique. Magnes. sulph.* ℥ss. *hodie.*

9th. Solution has been applied every other day. Is now quite well—indeed she has been so for two or three days. This was a severe case on its first appearance, and the result of course satisfactory.

Case 2.—*Catarrhal Ophthalmia of both Eyes, cured by the Ung. Argent. Nit.*

Mary Ann Lanthorne, æt. 53, seller of street breakfasts, 13th April, 1832. Slight catarrhal ophthalmia of left eye, not much vascularity nor epiphora; right affected, but to a less degree. Complaint has existed for six weeks.

Of rheumatic habit, and attributes the affection to cold in following her employment.

*Unguent. argent. nit. oculo utrique. Pil. hyd. Ext. col. c.* āā gr. v. *h. n.*

16th. Eyes improved.

*Pil. al. c. myrrh.* gr. v. *h. n. Repr. unguent.*

30th. Has absented herself since last report. Eyes much worse—great epiphora—agglutination of edges of palpebræ in the morning.

*Ung. hyd. nitrat. mitius palpebris. Pulv. rhœi, c. Cal. h. n. Mag. sulph. cràs. P. c. ung.*

May 2. Better. Excoriation at outer canthus of left eye, apparently from the epiphora.

*P. emp. canth. temp. sinistro.*

This plan was persevered in, and, on the 18th, she returned thanks and was dismissed. The only remnant of her malady, was a disposition to agglutination of the lids in the morning, and some weakness of vision. For this she had some dilute citrine ointment, and was directed to employ ablution, &c. Had the patient attended regularly in the first instance, it is more than probable that she would have been cured by two or three applications of the black ointment. As it was, the effect was marked and satisfactory.

CASE 3.—*Catarrhal Ophthalmia, of a Month's Standing, cured by Solut. Argent. Nit.*

George Marshall, a cabinet-maker, æt. 17, April 30th, 1832. Catarrhal inflammation of either eye—affection not very severe. Looks out of health—bowels confined. Affection has existed for a month—has only used warm water.

*Solut. argent. nit.* (gr. vj. *ad* ℥j.) *oculo utrique. Pulv. jal. c., C. mag. sulph. cràs.*

May 2d. Much better; scarcely any conjunctival redness. Sensation of sand between the globe and upper lid. *P.*

4th. Relapse last night. The weather is very cold, and many of the patients have applied with relapses.

*Pulv. cal. c. rheo, h. n. Mag. sulph. cràs. P. c. solutione.*

9th. Scarcely any epiphora or vascularity, but eyes feel weak in the mornings.

*P. c. solutione ter in hebdomadâ. Solutio hyd. oxymuriat.* (gr. j. *ad* ℥vj.) *pro collyrio, bis terve die.*

18th. Wishes to be discharged—merely a little weakness of the eyes in bright sunshine.

*Habeat solut. hyd. oxymur.* ℥vj.

Discharged.

CASE 4.—*Old Catarrhal Ophthalmia, cured speedily by Cup. Sulph.*

Martha Thorpe, æt. 29, March 28th, 1832. Catarrhal inflammation of one eye, of five months' duration.

*Pulv. jalap. comp.* ʒj. *Applicetur palpeb. Cup. sulph.*

30th. *Rep. Cup. sulph.*

April 11th. The blue stone has been applied every other day, and the patient considers herself cured. Dismissed.

We have taken these few cases of catarrhal ophthalmia indiscriminately and without selection, as they shew the usual method of treatment and its results. We have seen no case that has not terminated well and speedily, though, of course, we do not mean to say that catarrhal inflammation will not occasionally end in graver affections, under any management. However, it is quite certain that the copious leechings and active depletion that were formerly resorted to by all, and are still practised by many, are not to be compared, in point of safety and success, with the modern moderately-stimulating plan. As an instance of this, we may mention a case now under treatment. A woman applied, a few days ago, with old catarrhal inflammation of one eye and recent affection of the other. She stated that the inflammation of the former began just in the same manner with that of the latter. She had been from the first under the care of an eminent oculist; but, becoming dissatisfied at the progress of the case, she had just discontinued her attendance upon him. She had been freely leeched, cupped, and purged.

We dropped a solution of the argent. nit. into the worst eye, and vinum opii into that recently affected, applied a blister to the temple, and gave a strong aluminous lotion. Two days afterwards she returned, with the recent catarrhal inflammation cured, and that of the other eye much relieved.

Case 5.—*Chronic Inflammation of the Conjunctiva, rapidly cured by Solut. Arg. Nit.*

Eliza Cully, æt. 12, pauper, of very delicate appearance, May 9th, 1832. Considerable vascularity of the conjunctiva, both sclerotic and palpebral, of left eye—vessels on former disposed in form of net-work, of rather dull tint—palpebral conjunctiva villous in appearpearance. At outer margin of cornea, an elevation, apparently caused by deposition between the conjunctiva and sclerotica; slight pustules on this elevation, with contiguous superficial ulceration of margin of cornea. Little epiphora—slight intolerance—some nocturnal pain in temple—cornea clear—pupil rather contracted.

Affection has lasted three weeks—leeches and blisters have been applied —is getting worse rather than better. Was formerly O. P. twice.

*Pulv. rhœi, c. cal. h. n. Solut. arg. nit.* (gr. v. *ad* ℥j.) *ter in hebd. Ung. hyd. mitius o. mane palpeb.*

14th. Much the same.

*P. emp canth. ponè aurem.*

18th. Remarkable improvement—vascularity nearly gone. *P.*

23d. Cured—to have some citrine ointment.

As blisters had been previously applied without benefit, it is reasonable to suppose that the local stimulants were operative in effecting the cure.

II. Purulent Ophthalmia.

Case 6. *Gonorrhœal Ophthalmia, treated by the Unguent. Nigrum.*

John Durant, æt. 21, admitted Aug. 10th, 1831. The left eye is destroyed, nearly the whole of the cornea having sloughed away, and the humours having escaped—conjunctiva extremely vascular and chemosed—lids thickened but not much swollen—profuse purulent discharge—no pain.

Purulent inflammation commencing in right eye—conjunctiva and sclerotica not much inflamed—discharge slight—complains of pricking pain in the eye, which prevents sleep. Slight discharge from the urethra, with excoriation of the glans and inner surface of the prepuce. Health much impaired—general feverishness, with pains in the joints.

Has had gonorrhœa for the last two months. When gonorrhœa appeared, took Leake's pills for six weeks. The ardor urinæ and discharge diminished, but a fortnight ago the left eye became inflamed. He continued the pills for three or four days, but the eye becoming rapidly worse, he went to a medical practitioner, who applied four leeches to the temple; the leeches were repeated at short intervals to the number of twenty, and a blister was applied behind the ear. Two days ago the right eye became affected, and he was ordered by the same gentleman the same application of four leeches! He states that the disease commenced in the left eye in a manner precisely similar to that in which it appears in the right; the swelling of the lids and discharge came on about the fourth or fifth day, and gradually increased, attended with a throbbing pain, till the eye burst, when all diminished. The discharge from the urethra has gradually lessened since the left eye became affected. He is not aware of having transferred the gonorrhœal matter in any way to the eye; he had gonorrhœa once previously, but it was unaccompanied with any ophthalmic inflammation.

*Unguent. nigrum oculo dextro. Lotio aluminis oculo sinistro. Pulv. jalapæ comp.* ʒj.

11th. Says his right eye is quite free from the pricking sensation before experienced—redness not much diminished.

*To foment the eye with tepid water.*

12th. Inflammation of right eye much less, sight stronger, and he is surprised to hear that the application of the ung. nig. is again necessary. Discharge from left eye diminished. *P.*

14th. Inflammation now so trifling that the application of the ointment is omitted to day.

*Pulv. jalap. comp.* ʒj.

17th. Right eye nearly well—no pain in left.

*Guttæ argent. nit.* (gr. viij. *ad* ℥j.) *oculis ambis.*

Sept. 2d. Left eye has been gradually improving—right quite well.

CASE 7. *Gonorrhœal Ophthalmia cured by black ointment.*

James Griffiths, æt. 19, admitted Nov. 17th, 1831.

Both eyes inflamed—conjunctiva very vascular and slightly chemosed—corneæ and iris healthy, but sight dim—not much pain. Has had gonorrhœa for a fortnight, and the discharge from the urethra still exists, but has lately much diminished. The eyes have been hot, itchy, and uncomfortable for the last week, but inflamed for two days only; the inflammation rapidly becoming worse he came to this hospital.

*Ung. nigrum oculis ambis. Hyd. sub.* gr. vj. *c. Mag. sulph. postea.*

The ointment and pills were repeated on the 18th and 19th.

20th. Very much better—no remaining inflammation, save a slight increase of vascularity of the palpebral conjunctivæ—still slight discharge from the urethra.

*Omr. ung. nigrum. Guttæ arg. nit.* (gr. vj. *ad* ℥j.)

21st. *Rep. guttæ.*

23d. Well—dismissed.

CASE 8. *Gonorrhœal Ophthalmia—frequent Relapses—cured.*

John Haynes, æt. 21, admitted Nov. 27th, 1831. Conjunctiva and sclerotica of right eye exceedingly vascular, with slight chemosis—cornea dim with small ulcer in its centre—iris darker than that of other eye, but pupil regular and acting freely. Lids much swollen and inflamed, so that he cannot open the eye so widely as the left. Constant discharge of hot tears, but no purulent matter. Eye feels very hot and uneasy, but is free from pain—sight very dim. Slight discharge from the urethra without scalding.

Has had gonorrhœa for six weeks and taken copaiba. Inflammation of eye came on three days ago. He thinks it was caused by dirt getting into the eye, as the sensation was similar to what such would produce, but no extraneous substance could be found on examination. The inflammation has been gradually increasing.

*Ung. nigrum oculo. Hyd. sub.* gr. vj. *statim. Posteà Pulv. jal. c.*

28th. Eye feels much easier and cooler; epiphora diminished; redness much as yesterday—ointment caused pain for two hours.

*Rep. ung. c. pil.*

29th. Better—less redness—no pain.

*Rep. omnia.*

30th. Inflammation of conjunctiva much diminished—iris and cornea not improved in the same proportion—no pain—sight much clearer—bowels well open.

*Rep. omnia.*

Dec. 1st. Improving—cornea clearer—ulcer not so distinct—purged. *P.*

2d. Going on well. Cornea brighter—iris of natural colour—chief vascularity at margin of cornea. Eye-lids still much inflamed, and urethral discharge continues. *P.*

On the 5th the improvement was such that the ointment was discontinued, and the solution of nitrate of silver applied night and morning. On the 12th nothing remained but a slight inflammation of the palpebral conjunctiva. On the morning of the 13th the whole conjunctiva was found to be inflamed, with epiphora, but no pain.

*Ung. nigrum. Pulv. jalap. c.*

14th. No redness this morning; slight discharge of tears. *Resumr. guttæ argent. nit.*

19th. Came to-day with left eye inflamed. On the evening of the 17th he first felt the eye uneasy, the uneasiness rapidly increased, and now the conjunctiva and sclerotica are much inflamed—cornea and iris natural—heat and pricking pain in the eye.

*Ung. nig. Pulv. jal. comp.*

20th. Better—less inflammation—very little pain.

*Cal.* gr. vj. *h. n. Mag. sulph. cràs.*

23d. Not so well. Conjunctiva and sclerotica more inflamed—lids swollen—epiphora—eye feels hot and heavy.

*Ung. nigrum. Ant. tart.* gr. ij. *c. Mag. sulph.* ℥ss.

24th. Emeto-purgative operated.—Very little inflammation, save of palpebral conjunctiva.

*Cupri sulph. palpeb.*

We need not pursue the details farther. The patient was cured, but several slight relapses occurred.

These are all the cases of gonorrhœal ophthalmia entered in the books, as having been treated at the hospital within the last nine months. They are taken indiscriminately, and will serve, perhaps, to dispel the prejudices of those, who look upon the stimulating plan of treatment as a dangerous and unnatural innovation on the vested rights of leeches and phlebotomists. We can only judge of the merits of a particular mode of practice by comparing it with other modes. The most strenuous advocate of depletion could hardly choose a better text-book than the work of Mr. Lawrence on Gonorrhœal Ophthalmia. The amount of depletion resorted to in many of the cases treated and recorded by that gentleman is enormous. Patients were almost brought to the verge of the grave by the lancet, yet the inflammation of the eye went on, in too many to the destruction of the organ. It surely is a cutting satire on depletion, when its warmest advocate publishes a collection of cases in which it is employed with almost uniform want of success. If any gentlemen feel a doubt of the comparative safety and value of stimulants and depletion, we would recommend them to place side by side, and carefully compare, the foregoing cases reported from this hospital, with the long list of failures in Mr. Lawrence's work.*

Case 9.—*Ophthalmia Neonatorum of four weeks' duration, cured.*

Ann Jones, aged 5 weeks, Feb. 24th, 1832.

Purulent ophthalmia of right eye—lids slightly swollen and red, and pus pours out on their separation—cornea muddy, without ulceration. On the left eye a growth of conjunctiva overlapping the lower edge of the cornea.

Right eye affected four weeks. Left eye was affected at same time, but was relieved by the medical gentleman in attendance. Mother states that she was affected with leucorrhœa previous to delivery. *Ung. nigrum.*

*Alum lotion to be used with syringe.*

25th. Less inflammation—less discharge.

*P. ol. ric.* ʒj.

27th. Did not bring the child yesterday—doing well.

28th. Not so well.

*Omr. ung.—Appr. lot. argent. nit.* (gr. iv. *ad* ℥j.) *P. c. lot. aluminis.*

On the next day the discharge was completely gone, and the inflammation very slight. The lotion of the nitrate of silver was continued. On the 4th March, the child was discharged cured.

Case 10.—*Ophthalmia Neonatorum, of nearly five Weeks' duration, cured.*

Maria Lawrence, aged five weeks, March 9th, 1832. Purulent ophthalmia of both eyes—lids much swollen and inflamed—pus pours out on their separation—corneæ scarcely visible; they appear muddy, but without ulceration.

The nurse perceived discharge from the child's eyes a few days after its birth; but nothing appears to have been done. The mother had leucorrhœa at delivery.

*Unguent. nigrum. Lotio aluminis frequentius.*

On the 10th the child was not brought. On the 11th, the discharge from both eyes was less—lids still swollen.

*P. ol. ric.* ʒj.

12th. Discharge gone—scarcely any inflammation—an ulcer now seen on centre of cornea of right eye. *P.*

13th. Improving. Opens its eyes a little, and lids less thickened. *P.*

17th. Improving—right cornea still muddy.

*Solut. argent nit.* (gr. viij. *ad* ℥j.) *vice ung. nigri. P. c. Solutione aluminis.*

26th. Ulceration of the cornea healing. The child recovered perfectly.

---

* That work was very fully reviewed in this Journal, and our readers will find in our analysis the particulars of every, or almost every, case of gonorrhœal ophthalmia which it contains.

CASE 11. *Purulent Inflammation, with Ulcer of Cornea—Cure.*

John May, aged three weeks, Feb. 13th, 1832. Lids of both eyes much swollen—great purulent discharge from between them—intolerance of light, but can open the eyes a little. The cornea of right eye muddy and much ulcerated —that of left clear. Is stated to have had bad eyes since birth. For the first two or three days there was a discharge of scalding tears, afterwards of purulent matter.

*Unguent. nigrum. Lotio aluminis. Ol. ric,* ʒj. *cras mane.*

15th. Better—less discharge—ulcer of left cornea healing.

*Rep. ung. nig. et lotio et ol. ric.*

20th. Has not been here since 15th. Eyes better. Cornea of right eye clearing, and ulcer not so deep—left nearly well. Less discharge.

*Guttæ arg. nit.* (gr. vj. *ad* ℥j.) *o. mane. P. c. lot. aluminis.*

This patient, we believe did very well, and was dismissed soon afterwards cured.

CASE 12.*—*Gonorrhœal Ophthalmia.* George Taylor, æt. 21, admitted 26th Dec. 1831, with all the appearances of severe gonorrhœal ophthalmia in the right eye. The lids are red and swollen, and there is a plentiful discharge of thick purulent matter. On raising the lids the conjunctiva is seen red, and in a state of chemosis, so that the cornea is considerably below the level, and its edge is supposed to be slightly ulcerated —the centre remains perfectly clear. Some weeks ago—eight or nine—he remembers to have observed a discharge from the urethra, which produced no scalding or inconvenience; he took a few doses of salts, and in about ten days it disappeared. The urethra at the present time seems slightly inflamed, but there is no discharge observable. Nine days ago he felt a pain in his head, and if he stooped, or moved his head quickly, an intolerable sense of weight over the right eye, and the next day matter was discharged from it. On the third day he was unable to work, and an old woman recommended him to apply two leeches to the lower lid and a blister behind the ear, also to apply a cold poultice—by which he thinks the swellings of the lids was somewhat diminished—he also took salts and senna every day up to yesterday.

*Appl. c. c. tempor. ad* ℥xx. *Calomel,* gr. vj. *c. Opio* gr.j. *h. s. s. Magn. sulph.* ℥j. *cras mane. A lotion, with two grains of alum to the ounce, to be frequently injected.*

Mr. Guthrie turned out the thickened and vascular lids, and with a camel-hair pencil applied the black ointment freely over the whole surface of the inflamed conjunctiva, which appeared whitened after a few minutes by the application.

6, p.m. He has lost full ℥xx. of blood; the chemosed conjunctiva looks pale and flabby instead of the turgid and red appearance it had. The injection has been used, and the discharge appears less copious.

Dec. 27th. There is a sensible improvement in the case. The tumefaction of the lids has in a great measure subsided; the discharge is less, and he is free from pain; the chemosis is considerably reduced, and the conjunctiva has almost entirely lost its red and turgid appearance.

*Rep. magn. sulph. Cont. lotio. He has been freely purged.*

Dec. 28th. The chemosis has nearly subsided, the edges of the cornea are seen to be regular, the discharge is much diminished, and altogether there is the most decided improvement. The lids are still thick and vascular. He has used the lotion frequently, and has been freely purged by his medicine. The ointment has been applied to-day, but not quite so freely as before. Pills and salts repeated.

Dec. 29th. There is little perceptible change; he is going on well however. The black ointment has been applied.

31st. Continues improving.

Jan. 4th. 1832. He continues to improve. The swelling of the lids is abating, as is the inflammation. He is free from pain.

* This was not received sufficiently early to be incorporated with the others.

The black ointment has been applied.

6th. He is better; the redness of the conjunctiva seems to arise more from the lids having become granulated than inflammation. The black ointment has been omitted, and the sulphate of copper applied gently to both sides, with a drop of oil to each eye.

III. Strumous Ophthalmia.

There can be little doubt that many of the inflammatory affections of the textures of the eye are more or less modified by scrofula. But two particular affections would seem to be more frequently than others produced by this agency, or at least occur oftener in scrofulous subjects. These are the strumous inflammation of the cornea and its conjunctival covering, and the formation of little vesicles or pustules at the margin of the cornea. In both these affections, especially in the former, there is usually great epiphora and intolerance of light, these being in fact the most distressing symptoms. Yet we not unfrequently see the pustular or phlyctenular ophthalmia with scarcely any increase of lachrymation or intolerance of light. Such cases are managed without difficulty. In our present state of knowledge it would be an arbitrary and unjust decision to pronounce these the only scrofulous affections of the eye. Ulcers of the cornea, for instance, with very little corneitis, are sufficiently common in scrofulous children to justify us in suspecting the scrofulous habit as their cause. At present however we restrict ourselves to strumous corneitis and phlyctenular inflammation.

Case 13.—*Pustular Ophthalmia cured by the Oxymuriate Lotion.* Daniel Burns, æt. 5, April 9, 1832. This patient has been at the hospital formerly, but owing to some circumstance or other discontinued his attendance till the present time. He has now strumous ophthalmia of left eye, with marginal pustules. Vascularity not very great —much epiphora and intolerance. Catarrhal inflammation of right eye without pustules. Is a stout child, but is ill fed and has evident derangement of the digestive organs.

Left eye has been affected four days —right eye for a fortnight. Nothing has been done for him.

*Pulv. cal. c. scammoneâ,* gr. vi. *h. n. Mag. sulph. crâs. Lot. hyd. oxymur. sæpe indiès oculis. Ung. flavum.*

11th. Much improved. *P.*

16th. Merely slight intolerance. *P.*

30th. No pustules or phlyctenulæ—no epiphora nor intolerance. Merely a little weakness of right. To have some lotion. Dismissed.

We might detail several other cases in which a lotion of the oxymuriate or sulphate of zinc, or nitrate of silver, speedily removed this pustular affection. A moderately stimulating treatment with attention to the bowels will usually remove the present affection, but the tendency to relapse is not so easily got rid of. This can only be counteracted of course by what is calculated to improve the state of system upon which it depends.

Strumous corneitis is more severe, more mischievous in its effects, and more difficult to manage, than the preceding affection. It is singular, however, how instantaneously patients will frequently be relieved of all the acute symptoms. A patient will be suffering to the utmost degree, unable to open the lids or allow them to be opened, with deluges of scalding tears streaming down the cheeks, and considerable sympathetic fever, when suddenly under the effect of medicine these distressing symptoms will be dispelled as by a charm, and merely a little vascularity and weakness of the eye remain. Such patients, like the former, are very liable to relapse, and this tendency to sudden exacerbations and remissions it is, which has contributed to confer on particular modes of treatment, both empirical and professional, a degree of reputation which they do not in general deserve. At one time an emetic, at another an active purgative, or a blister, or hyosciamus, or calomel and opium, will achieve an almost miraculous cure to the astonishment of the patient and the great credit of the remedy. It is difficult, perhaps it is impossible, to point out the modifications which call for these various modes of treatment. In the present instance we shall merely record some cases illustrative of the characters of this singular complaint.

CASE 14.—*Strumous Corneitis, with Ulcers at Margin of Cornea—result not known.* Charles Angler, æt. 10, Dec. 21, 1831. Profuse flow of tears, not scalding, from right eye—great aversion to the light—on opening them near the light sneezing—some conjunctival inflammation—an ulcer on the inferior and inner margin of cornea, with red vessels running towards it. More conjunctival inflammation of left eye, the cornea of which is muddy and reddish—an ulcer at its outer and superior part. Patient of a strumous habit.

Right eye was affected with inflammation three years ago—present attack of three month's duration; it came on gradually.

*C. c. ad* ℥viij. *temporibus. Pulv. jalap. c.* ℈ij. *Appr. cup. sulph.*

26th. Better—less irritation. *P.*

28th. More intolerance of light and greater inflammation of conjunctiva.

*Emp. canth. temp. Ant. tart.* gr. ij. *pro emetico.*

30th. Much better, but still considerable inflammation.

*Cup. sulph. Pulv. jalap. c.* ℈ij.

Jan. 2d, 1832. Eye much better—corneæ more clear—vessels of conjunctiva less red. *P.*

4th. Not so well—more intolerance of light. *P.*

*A cloth wetted with one part of vinegar and ten of water to be laid upon the eye.*

6th. Better since application of lotion.

9th. A relapse, attributed by his mother to his having gone out in the damp.

*Emp. canth. temporibus.—Mag. sulph.* ℥ss. *Ant. tart.* gr. ij. *h. n.*

11th. Not any better.

*Hirud.* iij. *tempor. h. n.—Rep. Mag. sulph. c. Ant. tart. cràs.*

The patient was a little better at next visit, but the case is kept up no further.

CASE 15.—*Corneitis, with Marginal Pustule and Interstitial Deposition in the Cornea.*

Mary Lee, æt. 3, Jan. 28th, 1832. Conjunctival inflammation of left eye—pustule at inner margin of cornea, with red vessels running to it—muddiness of cornea—interstitial deposition, apparently commencing in centre—intolerance of light—excoriations about the lids, and vessels of the upper one enlarged.

Has had frequent attacks of inflammation of the eyes for the last four months. Present affection came on suddenly within the last week.

*Hyd. submur.* gr. ij. *Quininæ sulph.* gr. j. *M. 4tis horis sumend.*

21st. Conjunctival inflammation less—pustule at margin of cornea absorbed—cornea still muddy—interstitial abscess not increased. Child has rested better. *P.*

23d. Considerable conjunctival inflammation—interstitial deposition not increased.

25th. Conjunctival inflammation not increased—ulceration over the interstitial deposit, and a small quantity of matter in the anterior chamber; does not complain of much pain.

*Unguent. nigrum. P. c. Cal. et Quinin.*

26th. Eye very much improved—ulcer looks healthy—conjunctival inflammation much reduced, and part of the hypopium absorbed.

*Solut. arg. nit.* (gr. viij. *ad* ℥j.) *P. c. Hyd. sub. et Quininâ.*

27th. Ulcer healthy and conjunctival inflammation decreasing. *P.*

30th. Improved, and though medicine has not been given regularly, matter is becoming absorbed.

We need not follow up the case; under the foregoing plan of treatment it did well.

A very interesting case of strumous corneitis recently occurred. A scrofulous young girl, with strumous inflammation of the periosteum and bone of the right arm, had suffered, on several occasions, from inflammation of the eyes. She applied at the Ophthalmic Hospital with well-marked strumous corneitis of both eyes. She was cupped and purged, and the black ointment was introduced. On the succeeding visit she was improved, and the plan was persevered with. She then had a very severe relapse. The stimulating treatment was now abandoned, and emetio-purgatives, with cold-water lotions, were employed. The improvement was only temporary. The corneitis was as acute as we have ever seen it, the sympathetic fever considerable. Hyosciamus, in doses of five grains thrice daily, was next employed; it failed. The child was then put on calomel and opium. As soon as this affected her mouth, which it did in two or three days, the epiphora and intolerance disappeared, the vascularity and muddiness of the cornea subsided, and, from a state of great suffering, the patient was suddenly restored to one of comparative comfort.

In another case, which is still under treatment, a similarly fortunate result was obtained by an emeto-cathartic. The intolerance and epiphora in a male child were very great, so much so, that it was impossible to ascertain the exact amount of changes in the cornea. The child had suffered more or less from the affection for a year. At first, a solution of nitrate of silver, with blisters, were employed. These failed. The tartarized antimony, with Epsom salts, was then given. After the first dose, which operated strongly, the patient was able to open his eyes, the epiphora had disappeared, and hitherto no relapse has recurred.

We lately saw another case, under the immediate care of another gentleman. This appeared to have been a severe one, and we understand that several plans of treatment had been tried without success. This patient rapidly recovered under the administration of five grains of the extract of hyosciamus thrice daily. No doubt instances of this kind might be multiplied. In our next report, we shall endeavour to arrange the cases better than we are enabled to do at present, and to give such a full account of them as may prove serviceable to the professional public. Mr. Middleton has attached much importance to the sulphate of quinine. No doubt it is a valuable medicine, when given at the proper time; but it certainly will not effect with the patients at this hospital, what it appears to do with Mr. Middleton's.

Case 16.—*Corneitis, with Pustule—Interstitial Abscess.*

Henry Lee, æt. 11, Jan. 11th, 1832. General conjunctival inflammation of left eye—muddiness of cornea—interstitial deposition in its centre—varicose state of the veins of the upper lid. Intolerance of light—pain in the head and orbit—bowels regular.

Has been subject to frequent attacks of inflammation in the eyes. Present commenced suddenly, fourteen days ago, but has been much worse within the last five.

*C. c. temp. sinist. ad* ℥iv. *Hyd. submur.* gr. ij. 6*tis horis.*

12th. Much better. Can see the light without the degree of pain formerly experienced—vascularity diminished—less pain of the head and orbit.

13th. Cornea not so muddy, but ulceration commencing in it over the interstitial deposition—conjunctival inflammation less—intolerance of light much less—pain in eye and head diminished.

*Hyd. submur.* gr. ij. *bis die.*

16th. Mouth slightly affected—ulcer superficial, small, and healthy—inflammation less, and no pain.

*Omr. Hyd. sub. Pulv. jalapæ c.* ʒss. *Solut. argent. nit.* (gr. viij. *ad* ℥j.)

18th. Increased conjunctivitis.

*P. Appr. hirud.* iij. *tempori.*

20th. Inflammation of conjunctiva still considerable—ulcer healthy—pain of eye not increased—pain in the head. Leeches were not applied near enough to the eye.

*C. c. temp. dext. ad* ℥iij. *Hyd. sub.* gr. j. 6*tis hor.*

23d. Not much improvement.

*Hyd. sub.* gr. iij. 6*tis hor. Ung. argent. nit.*

27th. Improvement great, apparently from larger quantity of calomel—cornea clear, excepting over the interstitial deposition, where there is ulceration.

*P. c. Hyd. sub. Solut. arg. nit.* (gr. viij. *ad* ℥j.)

February 1st. Eye improved—cornea clearer—interstitial deposit almost wholly absorbed. *P.*

5th. Ulcer healing—conjunctival inflammation much less.

*Omr. Hyd. sub.*

℞. *Quin. sulph.* gr. ij. *bis die. P. c. Solut.*

15th. Cured and discharged.

IV. INTERSTITIAL ABSCESS.

CASE 17. R. Davis, æt. 2, admitted Jan. 17th, 1832. Interstitial abscess of the cornea, which has burst into the anterior chamber, at the bottom of which there is a small quantity of matter. The whole cornea is dim, and the abscess is situated in the centre, immediately in front of the pupil; the cornea at this point seems more prominent than natural, and ready to burst externally; the iris is very much discoloured, being reddish-brown, that of the healthy eye blue; conjunctiva and sclerotica a good deal inflamed. The child has been suffering, during the last month, under fever, accompanied with an eruption. The scales have only fallen off during the last week, and two or three red cicatrices may still be seen on the hand.

The eye was slightly inflamed before this eruptive fever; but, during its progress, the inflammation became much worse, the child appeared to suffer much pain, and would not allow the eye to be touched. At present, the little patient seems weak and irritable, but its mother says that, lately, her appetite is much improved—bowels regular. No treatment has been used to the eye.

℞. *Hyd. subm. Quinin. sulph.* āā gr. ss. *Sach.* gr. vj. *M. ft. pulv.* 4*tis h. s. Appl. Emp. canth. post aurem.*

18th. Opens the eye better—conjunctiva and sclerotica less vascular—quantity of matter in the anterior chamber rather increased.

*Omitt. quinin.*

℞. *Hyd. submur.* gr. j. 2*dis h. s.*

19th. Has taken six pills—mouth slightly affected. The eye is much less inflamed, and the child opens it wider, and will allow it to be touched: interstitial deposition rather less, and the matter in the anterior chamber diminished in quantity.

*Cont. Hyd. subm.* gr. j. 2*dis hor.*

20th. Cornea clearer—less matter in the anterior chamber—iris of a more natural colour—conjunctiva and sclerotica very slightly inflamed now. The child does not appear to suffer much. Mouth more sore.

*Cont. n. a.*

21st. Improving rapidly. External inflammation very slight indeed—cornea more transparent—interstitial deposit less—the quantity of matter in the anterior chamber much diminished—mouth more sore.

*Cont. Hyd. subm.* gr. j. 2*dis h.*

23d. Mouth not more affected by the mercury—a little more inflammation of the external tunics this morning—but the iris and cornea are more natural, and the quantity of matter in the anterior chamber so small, as only to be seen on very close examination.

*Hyd. subm* gr. j. 2*dis h.*

24th. Took 4 pills yesterday—soreness of mouth not increased—inflammation diminished.

*Hyd. subm.* gr. j. 6*tis h.*

25th. Mouth not more sore—eye much better—cornea more transparent, less deposition—no matter in the anterior chamber—very little redness.

*Cont. pil. No.* 4 *in die. Appl. Ext. belladonnæ, front.*

27th. Improving. Iris more dilated—took three pills yesterday—mouth rather more sore.

*Rep. pil.*

30th. Has taken four pills daily since the last date; the mouth, however, is not so sore as it was. The slight superficial ulceration of the cornea is healing. No matter in the anterior chamber—iris nearly of a natural colour—pupil rather contracted.

*Omit. Cal.*

From this period she took quinine as a tonic, and had the belladonna applied to dilate the iris.

V. ULCER OF THE CORNEA.

There appear to be several very distinct kinds of ulceration of the cornea, originating probably in different conditions of the system as well as of the part. It is very common in scrofulous children, and a great number of such cases are observed and treated at the Infirmary. In many there is a single circular ulcer of the cornea, usually in its centre, attended with little increase of vascularity, and not much epiphora or intolerance. There are several such cases at the hospital at present; and all are steadily advancing to recovery under the use of solutions of the nitrate of silver, or other stimulants, with attention to the bowels, and in some the sulphate of quina. Frequently the ulcer is productive of more inconvenience, and is either the sequel of severe catarrhal inflammation, strumous corneitis, or a pustule at the margin of the cornea. We have several interesting cases of extensive superficial ulceration of the cornea, but as they continue under treatment we shall defer their publication till the next report.

Case 18.—*Corneitis—Ulcer of the Cornea—result not determined.* Saunders Beavan, æt. 19, Jan. 25, 1832. Considerable conjunctival inflammation of the right eye—vessels very much enlarged, and branches shooting across the cornea—this is muddy—towards inner part of cornea a rather deep ulcer of an unhealthy appearance, with very irregular edges—much epiphora, but tears feel cold—pain in the head on stooping. Slight conjunctival inflammation of left eye. States that right eye has been affected for three weeks, and that it is gradually growing worse.

*Ung. nig. oculo dextro—Pulv. jalap. comp.* ℈i.

26th. Less pain—conjunctival inflammation and muddiness of cornea lessened—some enlarged vessels in left eye running towards margin of cornea.

*Ung. nig. oculis ambis. Mag. sulph.* ℥ss. *Ant. tart.* gr. j. *M.*

27th. Right eye much improved—ulcer looking healthy—left eye much benefited by the caustic—largest of the vessels destroyed.

*Rep. omnia.*

30th. Still considerable conjunctival inflammation of right eye—left much better.

*C. c. ad* ℥xij. *temp. dext. Ung. arg. nit. oc. dext. Solut. arg. nit.* gr. viij. *ad* ℥j. *oc. sinist. Rep. Mag. sulph. c. Ant. tart.*

31st. Left eye better—right improved, but ulcer not less. Admits that this eye has been inflamed for twelve months. *P.*

Feb. 3d. Ulcer diminishing.

*Cup. sulph. Pulv. jal. c.* ℈j.

This case proved very obstinate and tedious. The patient was placed on mercury, but with what effect is not stated in the hospital book. We detail this incomplete case, because its very unsatisfactory issue displays the real nature of the disease, and shews practitioners what they may expect. It is not fair to record merely instances of unusually rapid cure.

Case 19.—*Ulcer of the Cornea—Hypopium.*

Eliza Lait, æt. 3, Feb. 7th, 1832.

Small ulcer situated near centre of cornea of left eye—cornea more conical than natural—its whole surface dull and rough, and round its margin a kind of circular ridge, conveying the idea of the cornea having sunk below its natural level. Purulent matter in the anterior chamber. Inflammation of conjunctiva and sclerotica slight; red vessels being only discernible near the cornea, forming a pink zone around it, and some of them passing on to the cornea, and appearing to constitute the ridge alluded to. No pain, uneasiness, nor intolerance.

Eye has been inflamed three months. Six weeks ago the mother observed the first specks; and the second white spot at the bottom of the eye appeared three weeks afterwards. The eye has never been more inflamed than at present and the child has been so free from pain that the mother did not think it necessary to apply for relief.

*Hyd. sub.* gr. i. *Quin. sulph.* gr. i. *M. t. d. s.*

8th. *Om. Quinina. Cal.* gr. i. *duabus horis.*

13th. Mouth and gums slightly ulcerated—cornea clearer—ulcer smaller—quantity of matter in anterior chamber much diminished.

*P. c. Cal.* gr. j. *t. d.*

The case is not followed up in the book, but we understand that the patient did quite well.

A child was lately brought to the hospital with central circular ulcer of the cornea and hypopium; the ulcer was not over the hypopium, but the latter was mounting up towards the ulcer, and this was becoming deeper. It was almost feared that the eye could not be saved, or at least that the cornea would be penetrated by the ulcer and the usual consequences ensue. The child was put rapidly under the influence of mercury, and a solution of the nitrate of silver was at the same time employed for the ulcer. For two or three days the issue was doubtful. As soon as the system was fully under the influence of mercury, which in this instance it was, and perhaps to too great a degree, the hypopium almost suddenly disappeared. The ulcer seemed little affected by the mercury, but under the continued use of the solution of the nitrate of silver it was steadily filling up. The parents were alarmed at the salivation and deserted the hospital, but we have since ascertained that the patient recovered completely. As the details of the case were written on the letter and this was never presented by the parents, we are unable to relate the particulars more minutely. The leading facts, are as we state them, the child having been under our own immediate charge.

There is one circumstance to which we may allude before passing to the subject of iritis. Nitrate of silver produces discoloration of the eye, the whole surface of which assumes a dirty yellow tint, far from conducive to the personal appearance of the individual. So far as Mr. Guthrie knows, this has never occurred from the application of the black ointment, but always from solutions. This may, perhaps, be an argument in favour of the employment of the former.

The following case was treated with black ointment which had been made for two years; some person having expressed doubts of the efficacy of the ointment after having been kept for so long a period.

*Case* 20. William Mills, æt. 6 years, admitted Oct. 5th, 1831. Has an interstitial ulcer on the cornea with severe conjunctival inflammation of six months duration—he suffers great pain, and the tears are hot.

*Appl. Ung. argent. nit.*

℞. *Ant. tart.* gr. ii. *Lotio tepid.*

7th. Better—less inflammation, and does not complain of so much pain—can bear the light very well.

*Appl. Ung a. n. Rep. Ant. tart. et lotio.*

14th. Same.

17th. Better, ulcer has healed.

*Gutta argent. ung.* gr. viij. *ad* ℥j. *P. jalap. c.* ʒss.

19th. Much the same.

*Rep solutio.*

21st. Relapse—eye not so well, great intolerance of light and flow of tears.

*Appl. Ung. nig. a, a. P. jalap c.* ʒi.

24th. Better.

*Solutio argent.*

26th. Cured.

VI. IRITIS.

The three following cases of iritis were treated by the internal use of ol. terebinth.

CASE 21.—Maria Walbrook, æt. 45, admitted Dec. 2d, 1831. Left eye has been affected for six weeks, she then felt pain as if from a severe blow. The last two or three days there has been intolerance of light; sight very much impaired; pressure on the eye gives pain; subject to chronic rheumatism.

There is slight conjunctival inflammation, with a patch of vessels internal to the margin of the cornea; the pupil does not act on light, and is elongated obliquely upwards and downwards, and is irregular; the irregularity is near the inner portion, as if it arose from a partial separation; slight muddiness of the cornea; iris not much discoloured, but appears thicker than natural. This woman had a gonorrhœal discharge about eight months since; her bowels are regular.

℞. *Ol. terebinth.* ʒj. *3tiâ. q. q. h. s.*

3d. Eye is much improved, conjunctival inflammation less, and the pupil restored to nearly its natural figure, if the irregularity, as if from a portion being separated, is excepted; pupil does not act. The turpentine has produced slight giddiness and nausea.

*Cont. Ol. terebinth. Appl. lot. Ext. belladonnæ.*

4th. Her eye is improved; inflammation less; the pupil irregular and dilated; no intolerance of light; pain of the eye much less.

*Cont. terebinth. Lot. Ext. belladonnæ.*

5th. Eye better; pupil still dilated and irregular, and not acting; no pain.

*Cont. med. Omit. lot. Ext. belladonnæ.*

7th. Eye much better; sees more distinctly, but still the pupil is irregular and dilated; colour of the iris natural. Turpentine still causes giddiness and nausea.

*Cont. Ol. terebinth.*

9th. Increased conjunctival inflammation; the eye otherwise improved.

*Appl. hirudines*, No. iv. *Cont. Ol. terebinth.*

10th. Conjunctival inflammation relieved by the leeches.

*Cont. terebinth.*

12th. Sight improved, and iris of a natural colour, but the pupil does not act. *Cont.*

14th. Sight improved; pupil acts, but not so freely as it should do.

19th. Did not attend regularly between the 14th and this date. Eye is improved; sight better.

*Cont. terebinth. Appl. vin. opii.*

21st. Pupil acts more freely.

*Omit. terebinth. Cont. v. opii.*

This patient continued to attend for some time with rheumatic inflammation of the external tunics of the eye, but the treatment for the iritis terminated at this date.

Case 22. Anne Shea, æt. 42, admitted 4th Nov. 1832. This woman has been subject to frequent attacks of rheumatism. About three weeks since her left eye was attacked with dimness. She said it appeared as if a hair was across her eye, which would go off again; then it would seem like a fog or dimness. At that time the ball of the eye ached much, and there was a great flow of tears, which were hot. The eye has continued ever since to be dim, but the tears which now flow are cooler; not much intolerance of light; iris discoloured and irregular; pupil permanently dilated; redness of vessels round the cornea, which is slightly muddy; on looking through the pupil, perceive a greenish tinge; has not had any previous headach which she could refer to any particular spot. The chief pain she has felt in her head was last Summer, that appeared to be rheumatic and was general. Pulse 99, rather strong—skin hot—tongue white—bowels not open.

*Appl. C. c. post aurem ad* ℥xvj.

℞. *Pulv. jalap. c.* ʒj. *statim.*

℞. *Ol. terebinth.* ʒj. 4*tis h. s.*

5th. Inflammation much relieved by the cupping—pain less—bowels freely open.

*Cont. ol. terebinth.*

7th. The turpentine produced strangury and giddiness, so as to prevent its being given so frequently as directed, but the woman persevered. The eye is much improved.

*Cont. ol. terebinth.*

9th. Still greater improvement; pupil contracts on the stimulus of light, though not so much so as in a healthy state; iris less discoloured. The turpentine still keeps up great irritation—vision improved.

*Cont. ol. terebinth.*

11th. Still better—bowels confined.

℞. *P. jalap. c.* ʒj. *Cont. ol. terebinth.*

14th. Eye improved, pupil contracted and does not act; still the patient sees more distinctly—the irritation caused by the turpentine diminishing.

*Cont. ol. terebinth.*

16th. Iris of a natural colour; pupil contracted and motionless; no pain; sight improved.

*Omit. ol. terebinth. Appl. lot. Ext. belladonnæ.*

19th. Vision daily improves; still the pupil permanently contracted.

*Cont. lot. Belladon.*

23d. Sees more distinctly—no pain, but the pupil does not act.

*Lot. u. a.*

30th. Remains improving as to vision, but the iris motionless.

Dec. 26th. Did not attend again till this date, when her eye was quite natural in appearance; the pupil acted properly, and her sight was quite restored.

Case 23.—Thomas Clifton, æt. 32, admitted Dec. 14th, 1831. About 11 days since found weakness of the right eye, but he continued at his work till five days since—then, in consequence of the pain in the ball of the eye, he was obliged to leave off. At present there is great pain in the ball of the eye, increased upon pressure; intolerance of light; conjunctival inflammation, and considerable redness round the margin of the cornea, iris discoloured, pupil of a natural figure, but rather dilated and not acting upon light; upper eyelid hanging over the eye so as partially to close it. Cannot distinguish objects with the affected eye; has not had any syphilitic affection for some years, and is not subject to rheumatism—attributes the present attack to cold.

*Appl. C. c. temporibus ad* ℥xvj.

℞. *Ol. terebinth.* ʒj. *3tia q. q. h. s.*

15th. Relieved by the cupping.

18th. Pain less—pupil does not act—sight not much improved.

*Appl. C. c. ad* ℥xij. *Cont. ol. terebinth.*

17th. Pain much relieved, sees better, but still there is conjunctival inflammation; the pupil does not act; bowels regular; the turpentine produces slight giddiness.

*Cont. ol. terebinth.*

19th. Pain of the eye-ball less; conjunctiva still red; pupil dilated.

*Cont. terebinth. V.S. ad* ℥xvj.

21st. Pupil not so much dilated; pain nearly removed; none without pressure; conjunctival inflammation less; complains of irritation from the turpentine.

*Cont. terebinth.*

23d. Better.

*Appl. Ext. belladon. Cont. terebinth.*

26th. Pupil more dilated; iris less discoloured; sight better.

*Ol. terebinth. 6tis h. s. Appl. ext. belladon. bis die.*

30th. Conjunctival inflammation wholly subdued; sees better.

*Cont. med. et appl.*

Jan. 2d. Sees more distinctly.

*Cont.*

4th. Pupil more dilated, and those of the left eye affected by the use of the belladonna, so as to impair vision.

*Omit. belladon. Cont. terebinth.*

6th. Improving; pupil not so much dilated; vision improved; iris of natural colour.

*Cont.*

13th. Iris of a natural colour, and acts regularly; vision restored. Discharged cured.

The following cases of iritis were treated by the exhibition of calomel.

Case 24.—Thomas Down, æt. 61, admitted 1st Dec. 1831. He states that, on Tuesday week last, he experienced itching of the right eye, which was followed by dimness and pain, with flow of scalding tears, and great intolerance of light. The conjunctiva very vascular and red, the iris discoloured, and the pupil contracted and fixed; there is a patch of lymph clouding the inferior part of the iris. He has not had gonorrhœa or syphilis for 30 years. He complains of great pain on the slightest pressure.

*Appl. c. c. ad* ℥xvj.

℞. *Pil. hyd. subm. Ext. col. comp.* āā gr. v.

2d. Eye much the same.

℞. *Hyd. sub.* gr. ij. *Pulv. opii*, gr. ½. *M. secund. h. sumend. Appl. ung. hydrarg. palpabræ sup.*

3d. His eye is not so well; there is a greater deposit of lymph; the iris is more irregular, and the conjunctiva is more vascular.

℞. *Hyd. submur.* gr xxxvj. *Pulv. opii*, gr. vj. *M. et divid. in pil.* No xij. *una secund. hor. sumend.—rept. ung. hyd.*

4th. His eye is rather better; there is not so much deposit of lymph; less conjunctival inflammation; does not suffer so much pain; mouth slightly affected by the calomel.

*Rept. pil.* No. 1, *6tis h.—cont. appl. ung. hyd.*

5th. His eye is not quite so well; the deposit of lymph surrounds the pupil, but is thicker on its inferior margin, partly closing it. The conjunctiva is much the same.

*Rept. pil. 4tis h. s. Rept. ung. hyd.*

6th. His eye is better; there is not so much deposit; the conjunctiva is less inflamed; he complains greatly of his mouth, which is very sore; his bowels are much irritated and painful.

℞. *Ext. opii*, gr. ij. *statim. sumend.*

*Magn sulph.* ℥j. *cras mane.* *Appl. ung. hyd.* āā.

15th. Eye is much better, the deposit of lymph is nearly absorbed, the pupil is becoming more regular, and the iris is resuming its natural colour. He has not attended since the 6th, as his face was so much swollen.

℞. *Magn. sulph.* *Rept. ung. hyd.*

19th. His eye continues to improve; the deposit is less, and the inflammation has subsided. The pupil is still contracted and fixed, but the colour of the iris improves.

*Appl. belladon.*

21st. He continues to improve; the iris has resumed its natural colour, except at its under margin, where it adheres to the capsule of the lens, making the pupil contracted at that part—the superior portion of the iris being obedient to light.

*Appl. belladon.* ℞. *Magn. sulph.* ℥j.

23d. The iris adheres to the capsule of the lens, and the pupil remains contracted. The lens is getting opaque.

*Appl. belladonnæ.*

Jan. 4th. The iritis is cured; he merely attends at present for the application of the belladonnæ, to dilate the pupil.

Case 25. Martha Watson, æt. 27, admitted Jan. 3d, 1832. Two months back she had ulcer on the nates, buboes in the groin, and ulcerated sore throat, for which she took mercury for a week, and it produced ptyalism, when the chancre and the ulcers in her throat healed, and the buboes in the groin disappeared.

About 14 days since her left eye became very painful; her sight failed her, and there was great intolerance of light; and about eight days back she observed an eruption over her thighs and arms, the throat again ulcerated, and also the ulcer, which now discharges a little. She has applied eight leeches to the eye without benefit.

The conjunctiva is much inflamed, with a zone of vessels surrounding the cornea, the margin of which appears disposed to ulcerate, especially at its superior portion; the cornea is muddy; the iris discolored; pupil fixed and irregular, but not contracted; there is lymph clouding the inferior part of the iris, and great intolerance of light. She suffers acute pain, particularly on pressure, and there is a flow of scalding tears. There is a swelling on the forehead over the right eye, which gives great pain.

℞. *Hyd. subm.* gr. ij. 2*dis h. s.*

4th. Her eye is rather better; there is less pain and intolerance of light; the appearance of the eye unaltered. The spots upon the thighs and arms are large, copper-coloured, and scaly; they first came out as small, itching, and irritable pimples, and when they subsided, the eruption assumed the present appearance.

5th. Her eye is a little better; she is free from pain; the flow of tears has abated, and the intolerance of light is less.

The appearance of the eye not changed—her mouth is becoming sore.

℞. *Hyd. sub.* gr. ij. *Ext. opii,* gr. ss. *M.* 6*tis h. s.*

6th. She continues to improve; her mouth is sore.

℞. *Hyd. subm.* gr. iij. *Ext. opii,* gr. ¼, *M. nocte maneque sumenda.*

7th. She is much better; there is not so much conjunctival inflammation, and the flow of tears has ceased; the intolerance of light is much less.—Throat better, chancre healed up, and the swelling on the forehead has subsided. Her mouth still under the effects of calomel.

*Cont. pil. bis die.*

8th. Her eye improves, the lymph clouding the iris is not so thick as it was; the pupil is more sensible; throat nearly well; bowels affected by the medicine.

℞. *Hyd. subm.* gr. iij. *Ext. opii,* gr. j. *nocte maneque.*

9th. Continues to improve—mouth very sore.

℞. *Hyd. subm.* gr. iij. *Ext. opii,* gr. ¼, *nocte.*

11th. Idem. 13th. Still improves.

*Rep. pil.*

16th. She is much better, the eye being quite free from pain; her mouth is very sore; not any deposit of lymph; but the iris adheres to the capsule of the lens, at its inner and under portion.

*Omit. Hyd. sub. Appl. Belladonna.*

### VII. AMAUROSIS.

Among the cases entered under this head, is one where the patient could only distinguish one side of any object she looked at.

CASE 26.—Sarah Minching, æt. 27, was admitted April 13th, 1832. The patient states that, on awaking one morning, she was surprised to find she could only see the left half of an object; and that it made no difference which eye she looked with, or if she looked with both it was still the same. Her eyesight was perfectly good when she went to bed on the previous night. Works a great deal with her needle—had no unusual quantity of work before this attack—had no pain in the eyes or giddiness—catamenia regular. Was attacked with this affection of the eyes fourteen days since.

℞. *Magn. sulph.* ℥ss. *Ant. tart.* gr. ij. *quaque secunda nocte sumend.*

16th. States that she sees clearer; can see both the left and right side of an object, but the right is very dim and indistinct. Is much affected by a strong light. *Rep. med.*

18th. The dimness is still very great, and she states that her sight is no better than when last seen.

*Rep. Mag. sulph. et Ant. tart.*

23d. States her sight to be considerably better, and she can distinguish the right side of an object much more distinctly. In the evening she does not feel any inconvenience. *Rep. med.*

25th. Much the same as when last seen—sight not improved. *Cont.*

30th. Has continued the medicine since last report, and is now perfectly recovered—can see both sides of an object distinctly. Discharged.

### VIII. CATARACT.

Of the operations for cataract, two were for depression.

CASE 27.—Joseph Procter, æt. 22, Jan. 30th, 1832. Capsular cataract from injury when he was five years old. Mr. Guthrie separated the capsule from its adhesions, and depressed it. Slight attack of inflammation followed, which the usual antiphlogistic remedies overcame; and, on the 4th of February, he was discharged, the cataract having become almost entirely absorbed, and since that period has been completely.

CASE 28.—Edward Collins, æt. 13 months, May 21st, 1832, with capsulo-lenticular cataract of the right eye.

Mr. Guthrie depressed the cataract, and the case did well—was discharged from hospital the following day, and has since gone on very well.

There have been six patients operated on for breaking up the cataract.

CASE 29.—James Stockman, æt. 25, Jan. 27th, 1832. Capsular cataract of the right eye, first noticed ten months since.

Mr. Guthrie separated the adhesions of the capsule with the iris, and broke up the cataract. He was discharged on the 1st Feb. The capsule still seen floating, but it was ultimately absorbed without further operation.

CASE 30.—Edward Madle, æt. 25, Jan. 27th, 1832. Capsulo-lenticular cataract of the left eye, the right eye being perfectly sound. He did not notice the gradual formation of the cataract.

Mr. Guthrie broke up the cataract; partial absorption took place. On the 29th Feb. he was again operated upon; much more of the lens became absorbed after this; but, May 20th, it was necessary again to operate, and the capsule was more freely broken up, and a slight adhesion, which it still had with the iris, separated. The cataract, after this, became so absorbed as not to interfere with vision.

During this last operation, when Mr. Guthrie separated the capsule from the iris, the iris, at that portion, instantly became changed in colour, becoming quite green, and this, on the following day, had spread over the whole iris; so that, as to colour, it had the appearance of acute inflammation.

CASE 31.—Ellen Mills, æt. 15; 1st February, 1832. Soft cataract of both eyes.

Mr. Guthrie operated on the right eye by breaking up the lens. After the operation, a considerable portion was absorbed; but it required that the lens should be further broken up, and, on 29th Feb. the operation was repeated.

On the 5th March she was discharged, and afterwards attended a short time as an out-patient—the lens was completely absorbed.

Case 32.—Thomas Sewiss, æt. 10, February 1st, 1832, with congenital cataracts of both eyes. His father stated that his sight was first affected seven years ago, but that it was only two years since he became quite blind. His mother's father brought him to the hospital, and was affected in a similar manner in both eyes.

Mr. Guthrie broke up the cataracts in both eyes. The cataracts did not appear to have been much absorbed after this operation; therefore, on the 29th February, the operation was repeated in both eyes, after which much more of the lens in each eye became absorbed. But still portions of the hardened capsule required a third operation in either eye; but this was quite successful, the boy's vision being restored by the complete absorption of the cataracts.

Case 33.—Jane Brazier, æt. 5, May 5th, 1832. Congenital cataract of both eyes.

The operation for breaking up the lens and capsule was performed in both eyes. Slight constitutional irritation succeeded the operation, but no local inflammation; she was discharged in a few days, absorption of the cataracts going on rapidly *(see W. Hoole's case)*.

There were six operations for extraction of the lens.

Case 34.—Mary Sullen, æt. 65, 1st February, 1832. Hard cataract of the left eye—the right lens is also becoming opake.

Mr. Guthrie extracted the lens, making the section of the cornea upwards. Slight inflammation followed the operation, which was subdued easily; and, on the 6th February, the eye was quite free from inflammation, cornea had united smoothly, pupil regular, and can see with the eye very well.

Case 35.—Sophia Williams, æt. 60, 29th February, 1832, with hard cataracts of both eyes. Cannot see any thing with the right, and but faintly with the left. The cataract was removed from the right eye, by extracting the upper section of the cornea. There was, for several days, some inflammation, but this was subdued, and the eye became quiet, and she was discharged 20th March, and could see pretty well.

Case 36.—Charlotte Richards, æt. 45, March 14th, 1832. Cataract of both eyes, the left more advanced, and Mr. Guthrie removed the hardened lens in the usual manner; the usual after-treatment was adopted, and she was discharged 24th March; but her sight was rather imperfect, on account of chronic conjunctival inflammation.

Case 37.—James Stovey, æt. 72, April, 25th, 1832. Had cataract of the right eye. The operation was performed in the usual manner; but on the 2d May, after having gone on well till then, great inflammation was set up from cold; he is now (June 1st) in Hospital, the cornea having suffered from the inflammation, and being opake.

Case 38.—Hariet Highfield, æt. 58, May 4th, 1832. Hard cataract of the right eye—lens of the left becoming opake. The right lens extracted in the usual manner. There was very little after-treatment necessary, the local and constitutional symptoms being mild, and on the 13th she was discharged cured.

Case 39.—David Silver, aged 68, admitted 19th March, 1832, with hard cataract of the left eye, and incipient cataract of the right.

On the 23d, Mr. Guthrie performed the operation of extraction in the usual manner. The operation was succeeded by violent inflammation, and although the lancet was freely used, suppuration ensued. The patient did not complain of any pain during the inflammation.

The next case should have been inserted with those where the cataract was broken up.

Case 40.—W. Hoole, æt. 67; March 2d, 1832. Mr. Guthrie broke up a capsular cataract of the left eye, which the patient had noticed only for six months; then followed considerable fever and local inflammation, and, on the 28th of May, the report states there is still slight conjunctival inflammation; the pupil is clear, and his sight improving daily. He was then discharged.

CASE 41.—There was one case of milky cataract. The patient, a man of the name of Evan Davies, was operated upon on the 2d March, 1832; but he will require a further operation before he recovers the use of that eye.

We conclude with a list of the patients who have attended at the Institution from the 1st January to the 1st of June; their diseases are specified as well as the numbers in the different months. We shall endeavour in future to give a Quarterly Report from this useful Institution, and to render it as accurate and conducive to the dissemination of ophthalmic knowledge as possible.

REPORT of the PATIENTS attending the ROYAL WESTMINSTER OPHTHALMIC HOSPITAL, from January 1st to June 1st, 1832.

| DISEASES. | Jany. | Feby. | March. | April. | May. | Total. |
|---|---|---|---|---|---|---|
| Amaurosis | 3 | 11 | 7 | 11 | 6 | 38 |
| Amblyopia | .. | 4 | 3 | 8 | 4 | 19 |
| Cataract | 2 | 7 | 7 | 4 | 8 | 28 |
| Chemosis | 1 | 1 | .. | .. | .. | 2 |
| Catarrhal Inflammation | 15 | 24 | 22 | 40 | 41 | 142 |
| Cornea, Inflammation of | 2 | 5 | 2 | 6 | 6 | 21 |
| ———, Ulceration of | 9 | 6 | 5 | 2 | 4 | 26 |
| ———, Pustule of | 6 | 5 | 1 | 2 | 4 | 18 |
| ———, Opacity of | 1 | 2 | 5 | 1 | 3 | 12 |
| ———, Chronic Inflammation of | 3 | 3 | .. | 1 | 4 | 11 |
| ———, Specks on | 5 | 10 | 10 | 11 | 12 | 48 |
| ———, Conical | .. | 1 | .. | .. | .. | 1 |
| Conjunctival Inflammation | 10 | 23 | 22 | 16 | 12 | 83 |
| Chronic Ophthalmia | 1 | 1 | 1 | 5 | 3 | 11 |
| Disorganization | 2 | 4 | .. | 1 | .. | 7 |
| Eye-Ball, Protrusion of | 1 | .. | .. | .. | .. | 1 |
| Eye-Lids, Inflammation of | 8 | 19 | 17 | 11 | 14 | 69 |
| ——— Tumour of | 2 | 1 | .. | 4 | 2 | 9 |
| Iris, Inflammation of | 3 | 2 | 7 | 1 | .. | 13 |
| ——, Prolapsus of | 1 | .. | .. | .. | 1 | 2 |
| Glaucoma | 1 | 1 | .. | 2 | 1 | 5 |
| Hypopium | .. | 1 | .. | .. | .. | 1 |
| Choroid Coat, Inflammation of | .. | .. | 1 | .. | .. | 1 |
| Lachrymal Sac, Inflammation of | 1 | .. | 1 | .. | .. | 2 |
| ——— Duct, Obstruction of | 2 | .. | .. | 3 | 3 | 8 |
| Lens, Prolapsus of | .. | .. | 1 | .. | .. | 1 |
| Interstitial Abscess of Cornea | 3 | .. | 1 | 1 | 2 | 7 |
| Pustular Inflammation | 3 | 12 | 13 | 1 | 3 | 32 |
| Purulent Ophthalmia | 2 | 4 | 5 | .. | 2 | 13 |
| Sclerotic Coat, Inflammation of | .. | .. | 1 | .. | .. | 1 |
| Staphyloma | 1 | 3 | 1 | 1 | 6 | 12 |
| Strumous Ophthalmia | 2 | .. | 1 | 9 | 10 | 22 |
| Varicose Veins of the Orbit | 1 | .. | .. | .. | .. | 1 |
| Injuries to the Eye | .. | .. | .. | 6 | 8 | 14 |
| Affection of Portio Dura | .. | .. | .. | 1 | .. | 1 |
| Pterygium | .. | .. | .. | 1 | .. | 1 |
| Strabismus | .. | .. | .. | 1 | .. | 1 |
| TOTAL | | | | | | 684 |

### LXXXVI.

### Cholera in the Ship Brutus.

The outbreak of the epidemic in the above unfortunate vessel, exceeds in fatality any thing that has yet occurred in England. A vessel of about 300 tons contained 330 passengers, most of whom were in a state of great poverty, being ill clothed, and not having provisions for half the voyage, was attacked with the epidemic on the 10th day (one account says the 8th) after sailing from Liverpool; and, in a few days, 81 out of 117 fell victims. The passengers were entirely paupers, and it appears that the weather, when the epidemic was at its height, was hazy and tempestuous. The state of crowding, the condensation of human effluvia, the scanty food, and the great deficiency of clothing, account, in a great measure, for the fatality of the attack. Those only who have seen ships with emigrants for America, can form an idea of the misery and crowding, and bad air at night, when the hatches are closed, and the people all in the 'tween decks. It required, however, an epidemic influence to render such a place a lazar house.

---

### LXXXVII.

### The late Dr. Dill.

It is with deep regret we announce the death of this talented and zealous young physician, cut off in the flower of his age and faculties, and while contributing largely to the extension of science and the diffusion of knowledge by his active mind and pen. Dr. Dill was the son of a highly-respectable clergyman in the county of Derry, and a graduate of Edinburgh. He had been several years resident medical officer in the Fever Hospital, pursuing his clinical and physiological researches with great ardour, and contributing largely to many periodical and other works, though his name seldom appeared. During the last four years, Dr. Dill had a regular engagement in the review department of this Journal. The last article from his pen, was the extended review of Dr. Bright's "Reports of Medical Cases."

The profession is aware that Dr. Dill took an active and zealous part in the investigation of the epidemic cholera in this metropolis—the more so as he was the author of a distinguished article on that subject in the Foreign Quarterly Review, where he gave a fair statement of the case, though leaning somewhat to the doctrine of contagion. In the midst of this investigation, Dr. Dill was seized with typhoid, or rather petechial fever, caught, apparently, from some patients labouring under the same disease in the hospital; and, although every effort was used by his medical friends and attendants, this amiable and promising young physician died on the 15th day from the commencement of the attack. We, who had ample means of knowing Dr. Dill's character and attainments, have the deepest cause to mourn his premature death! His labours are terminated, but his memory will long be cherished by all those who had the pleasure and advantage of his acquaintance.

---

### LXXXVIII.

### Medical Constitution.

Our observations induce us to think that a gastro-enteric diathesis or constitution is now forming in this country, or, at all events, in this metropolis, similar to that which commenced about the same period (June) of last year—and that, if heat and moisture alternate, as of late, we shall probably have another brush of cholera or cholerine, this Summer and Autumn. We have observed a preternatural irritability of the alimentary canal, for some weeks past, in consequence of which, slight errors of diet, exposure to cold, or even mental perturbation, bring on diarrhœa, ending occasionally in well-marked cholera, so called. Within these few days two individuals died in the Haymarket, presenting all the features of the recent epidemic. One of them (a female) had not been out of her room for many months previously. She was seized, after eating food of difficult digestion, with diarrhœa, which, in 24 hours, rose to cholera, and killed her in 12 hours. The other (a foreigner) ate heartily of pease-soup and mackarell—was seized with sickness and purging, ending in fatal collapse. Even an irritating dose of physic, in health, risks an attack of cholera. We trust that, should the epidemic again become prevalent, our public institutions will not be shut against the sick poor. Our prejudices on this head have made us the laughing-stock of enlightened Europe.

*22d June*, 1832.

LXXXIX.

Dr. Seeds' Ophthalmic Lotion.

Since our last, Dr. Seeds has shewn us some cases in which he used this lotion, the composition of which is mentioned in our last Number. In all it was productive of much benefit, particularly in the instance of a gentleman who had received a wound in the eye in Albania, and who had partial staphyloma.

Dr. S. is now in his 77th year, and we are sure that the benevolent feeling which prompts him to devote much time and trouble to the employment of a remedy which he believes to be advantageous, cannot fail of obtaining admiration.

